# Rearing of *Mallada basalis* (Neuroptera: Chrysopidae) on modified artificial diets

**DOI:** 10.1371/journal.pone.0185223

**Published:** 2017-09-29

**Authors:** Jingwen Ye, Jun Li, Zhigang Li, Shichou Han

**Affiliations:** 1 Department of Invasive Biological Control, Guangdong Institute of Applied Biological Resources, Guangzhou, Guangdong Province, the People’s Republic of China; 2 Guangdong Public Laboratory of Wild Animal Conservation and Utilization, Guangzhou, Guangdong Province, the People’s Republic of China; 3 Guangdong Key Laboratory of Animal Conservation and Resource Utilization, Guangzhou, Guangdong Province, the People’s Republic of China; Chinese Academy of Agricultural Sciences Institute of Plant Protection, CHINA

## Abstract

*Mallada basalis* (Walker) has the potential to be a valuable biological control agent because of its predatory abilities, strong reproductive capacity, and broad prey range. This study aimed to improve on a previously used artificial diet for *M*. *basalis*, to achieve a longer oviposition period and greater survival rate and fecundity. We analyzed the development, survival, longevity, and reproduction of *M*. *basalis* (F1 and F2 generations) fed two artificial diets (AD1 and AD2). Both diets contained chicken egg yolk, beer yeast powder, honey, trehalose, seawater spirulina, and potassium sorbate. AD1 also contained sucrose and vitamin C. The duration of F1 1st larvae, F1 2nd larvae, F1 pupae, F2 egg, and F2 2nd larvae reared on AD1 were significantly shorter than those reared on AD2. F1 adult longevity and F2 oviposition period for AD1 (45.40 d and 31.00 d) were significantly longer than for AD2 (30.74 d and 20.80 d). All the following were significantly greater for AD1 compared with AD2: F1 female proportion, F1 daily oviposition, F1 female oviposition, F2 daily oviposition, F2 female oviposition, F1 emergence rate, F2 pupation rate, and F3 egg hatching rate. Moreover, for *M*. *basalis* fed AD2, the duration of F2 2nd and 3rd larvae (9.00 d and 8.64 d) were significantly longer than for F1 (4.70 d and 4.92 d). The F1 oviposition period (31.57 d) was significantly longer than F2 (20.80 d). The F2 female oviposition (189.20 egg/female) was significantly less than F1 (307.14 egg/female). We found that the oviposition period and female longevity of F1 reared on AD1 was longer than that reared on the artificial diet in a previous study. The daily oviposition and female oviposition of F1 from AD1 was larger, while the F2 egg hatching rate was greater compared with that from the previous diet. However, the offspring of *M*. *basalis* fed AD2 were less thrifty. We found diet AD1 supported development and reproduction better than AD2 and the diets in our previous study. These findings may contribute to the mass rearing of this economically important predatory green lacewing.

## Introduction

Green lacewings (Insecta: Neuroptera) are important predators; 1350 species of 86 genera are known worldwide. The insect is abundant in China, with about 100 species of 15 genera recorded. They are one of the most important predators for pest control in agriculture and forestry because of their wide distribution, large population, and extensive range of prey species [1–4]. *Mallada basalis* (Walker) (Neuroptera: Chrysopidae) is a common predator of natural fauna in agricultural fields in Hainan, Guangdong, and Taiwan, China [5–7]. The larvae of this lacewing are general predators, whereas the adults feed on nectar, honeydew, and pollen [8]. Previous investigations suggest its potential as a biological control agent against several species of pests, including *Phyllocnistis citrella* Stainton, *Aphis* spp., *Nipaecoccus filamentosus* (Cockerell), *Diaphorina citri* Kuwayama, and *Panonychus citri* on citrus; *Aphis gossypii* Glover on sweet pepper; *Tetranychus urticae* Koch and *Tetranychus kanzawai* on strawberry; *Planococcus citri* on Indian jujube; and *Icerya aegyptiaca* (Douglas) on several tree species [6, 9–10]. Moreover, they are successful predators, have strong reproductive capacity [6–7, 11]. Research has demonstrated that *M*. *basalis* has some tolerance to selected insecticides, fungicides, and acaricides [12, 13].

As environmental issues increase, environmentally friendly approaches have become important in agricultural pest management. Biological control, which utilizes carefully screened/selected natural enemies to suppress pest populations, is considered environmentally safe and a viable alternative to pesticides [14]. For this to succeed, the mass production of their natural prey, or a substitute living prey, is needed for them to feed on during the captive rearing stage [13]. In an augmentative biocontrol strategy, large quantities of beneficial insects and mites are reared and released onto the crop. A conservative method for mass rearing these natural enemies is via a so-called natural, tri-trophic system, comprising the predator (parasitoid), the herbivorous prey (host), and the prey’s host plant [15]. However, this is often time consuming and/or expensive. The availability of an effective artificial diet addresses these problems, shortens the production line, and consequently represents a step towards more cost-effective mass rearing [16]. The availability of effective factitious food sources should reduce the number of trophic levels, resulting in lower costs for labor and production facilities, such as greenhouses [15, 17]. Artificial diets have been developed to sustain the mass rearing of a wide range of arthropod natural enemies with varying success [18]. In previous studies, we found an artificial diet supported development and reproduction of *M*. *basalis* [19]. The main objective of the current study was to improve the artificial diet to obtain a longer oviposition period, a greater survival rate, and to improve the fecundity of *M*. *basalis* for the mass rearing of this economically important predator.

## Materials and methods

### Experimental insects

*M*. *basalis* larvae were collected in 2010 from guava (*Psidium guajava* L.; Myrtales: Myrtaceae) trees in a guava orchard in Wenchang City, Hainan Province, P.R. China. The owner of the orchard gave permission for the study to be conducted on this site. *M*. *basalis* larvae were reared in the laboratory on a diet of eggs of the rice grain moth, *Corcyra cephalonica* [20]. The *C*. *cephalonica* eggs, which had been laid by females reared on rice bran, were irradiated with ultraviolet light for 30 min to kill the embryos before being offered as food for the *M*. *basalis* larvae [21]. The *M*. *basalis* culture was reared at 26 ± 1°C, 70 ± 5% RH, and a 16:8 h L:D photoperiod. The 21st generation of *M*. *basalis* was chosen for experiments, and for purposes of the present study, this 21st generation was designated as the parental generation, P. Their eggs and subsequent life stages were designated as the F1 generation. Likewise the eggs of F1 (F2) females and subsequent life stages belonged to the F2 (F3) generation, respectively.

### Preparation of artificial diets

The artificial diets were formulated based on that described by Ye et al. [19]. Two artificial diets (AD1 and AD2) were prepared for the *M*. *basalis* larvae, and their composition, as well as the composition of the previous artificial diet (AD0) are shown in Table 1. Compared with our previous work, seawater spirulina was added to both AD1 and AD2 as a dietary protein source [22]. In addition, sucrose and vitamin C were removed from AD2 to determine whether they were essential ingredients. If not essential, their subsequent removal from the diet would reduce food costs. Diet ingredients were blended in a food processor for 3–5 min until the entire mixture was homogenous. A 10 × 10 mm Parafilm membrane was stretched to about 3 × its original length and width [23]. The diet was placed centrally on the membrane which was folded and stuck tightly together. The artificial diets were prepared every 2 weeks and kept in a refrigerator at 5°C. The weight of each diet packet was 0.05 g, while the weight of the diet itself was 0.03 g.

**Table 1:** Composition of three diets for rearing larvae of the chrysopid *Mallada basalis*.

Ingredients	Ingredient amount (g)		
	AD0	AD1	AD2
Chicken egg yolk	40	40	40
Beer yeast powder	30	30	30
Honey	20	20	20
Sucrose	9	9	0
Trehalose	1	1	1
Seawater spirulina	0	1	1
Vitamin C	0.1	0.1	0
Potassium sorbate	0.1	0.1	0.1

Acronyms: AD0, previous artificial diet, AD1, artificial diet 1, and AD2, artificial diet 2

### Experimental setup

*M*. *basalis* neonate larvae (n = 50; F1 generation) were counted into Petri dishes (90 mm diameter × 15 mm height), one for each treatment. Each neonate larva was placed on the bottom of the Petri dish. In both treatments the larvae were supplied with five diet packets and water in moist cotton every day. The duration of the development of each life stage from the neonate to the cocoon were determined by monitoring molting events every day. Mortality was recorded each day during development of the immature stages. These experiments with the F1 generation were continued until the females died, and at that time, adult longevity was recorded. Dead males were replaced by males of similar age from the laboratory colony to ensure females were mated.

After the F1 generation adults had emerged, their sex was recorded, and then each adult female was paired with a male. The F1 adults were fed brewer’s yeast powder, honey solution (honey to water = 1:2 by volume), and water in a moist cotton wad. The fecundity of each F1 female (eggs laid per day) was recorded. Ten Petri dishes, each with the eggs of a single female were used for each treatment in order to determine the hatching percentage from the eggs of the F2 generation. There were dozens of eggs in each Petri dish, and 10 of them were used for measurement of hatching rate. The eggs were collected on the seventh day after laying [24]. This procedure was repeated five times. Experiments were carried out as above in a growth chamber at 26 ± 1°C, 70 ± 5% RH, and a 16:8 h L: D photoperiod until F3 eggs hatched.

### Data analysis

Data were subjected to statistical analysis using SPSS 17.0 software (SPSS Inc., Chicago, Illinois, USA). Differences in larval and pupal developmental duration, developmental parameters, and reproduction were analyzed by paired *t*-test. These analyses were performed on data of five replicates per treatment.

## Results and discussion

The duration of 1st larvae, 2nd larvae, and pupae of generation F1 reared on AD1 (3.94 d, 4.27 d, and 9.25 d, respectively) were significantly shorter than those reared on AD2 (4.54 d, 4.70 d, and 10.46 d, respectively; paired *t*-test, *t* = -2.48, 2.13, and -4.28, respectively; df = 49, 43, and 23, respectively; *P* < 0.05; Table 2).

**Table 2:** Developmental duration of the immature stages of F1 generation *Mallada basalis* fed on two artificial diets.

Diet	Duration of development (d)			
	1st instar	2nd instar	3rd instar	Pupa
AD1	3.94 ± 0.13b	4.27 ± 0.16b	4.62 ± 0.32a	9.25 ± 0.20b
AD2	4.54 ± 0.21a	4.70 ± 0.23a	4.92 ± 0.29a	10.46 ± 0.23a

Means (± SE) within a column followed by the same letter do not differ significantly (paired *t*-test; *P* > 0.05). Acronyms: AD1, artificial diet 1, and AD2, artificial diet 2

The F1 preoviposition period in the AD1 treatment was 7.57 d, which was significantly shorter than that in the AD2 treatment at 12.14 d (paired *t*-test, *t* = -3.60, *df* = 4, *P* < 0.05). The F1 adult longevity from AD1 (44.57 d) was significantly longer than that from AD2 (30.74 d; paired *t*-test, *t* = 0.99, *df* = 6, *P* < 0.05). The F1 female proportion in the AD1 treatment (0.56) was significantly larger than that in the AD2 treatment (0.36; paired *t*-test, *t* = 2.78, *df* = 4, *P* < 0.05). The F1 daily oviposition in the AD1 treatment (17.57 eggs/female/day) was significantly larger than that in the AD2 treatment (8.69 eggs/female/day; paired *t*-test, *t* = 2.24, *df* = 6, *P* < 0.05). The F1 female oviposition from AD1 (481.29 eggs/female) was significantly larger than that from AD2 (307.14 eggs/female; paired *t*-test, *t* = 1.05, *df* = 6, *P* < 0.05; Table 3).

**Table 3:** Reproduction and oviposition parameters of the F1 adult progeny of *Mallada basalis* on the two artificial diet treatments.

Parameter	Diet	
	AD1	AD2
Preoviposition period (d)	7.57 ± 1.25b	12.14 ± 1.87a
Oviposition period (d)	32.86 ± 6.07a	31.57 ± 6.05a
Female longevity (d)	45.40 ± 13.54a	49.20 ± 6.24a
Average longevity of females plus males (d)	44.57 ± 7.24a	30.74 ± 5.99b
Female proportion	0.56 ± 0.06a	0.36 ± 0.05b
Daily oviposition (eggs/female/day)	17.57 ± 3.74a	8.69 ± 2.14b
Female oviposition (eggs/female)	481.29 ± 54.40a	307.14 ± 60.77b

Means (± SE) followed by the same letter within a row do not differ significantly (paired *t*-test; *P* >0.05). Acronyms: AD1, artificial diet 1, and AD2, artificial diet 2

The F1 emergence rate in the AD1 treatment (94.00%) was significantly greater than that in the AD2 treatment (65.00%; paired *t*-test, *t* = 4.26, *df* = 4, *P* < 0.05). There was no significant difference in the F1 pupation rate and F2 egg hatching rate between the two diet treatments (paired *t*-test, *t* = -0.78 and 1.63, respectively; *df* = 4; *P* = 0.48 and 0.18, respectively; S1 Fig).

The duration of eggs and 2nd larvae of F2 fed AD1 (3.28 d, 5.38 d, respectively) were significantly shorter than those fed AD2 (4.52d, 9.00 d, respectively; paired *t*-test, *t* = -12.77 and -4.36; *df* = 49 and 31; *P* < 0.05; Table 4). The F2 oviposition period in the AD1 treatment (31.00 d) was significantly longer than that in the AD2 treatment (20.80 d; paired *t*-test, *t* = 1.00, *df* = 4, *P* < 0.05). The F2 female proportion in the AD1 treatment was 0.55, which was significantly larger than that in the AD2 treatment at 0.75 (paired *t*-test, *t* = -0.88, *df* = 3, *P* < 0.05). The F2 daily oviposition from AD1 (15.56 eggs/female/day) was significantly larger than that from AD2 (6.80 eggs/female/day; paired *t*-test, *t* = -0.39, *df* = 5, *P* < 0.05). The F2 female oviposition in the AD1 treatment (456.40 eggs/female) was significantly larger than that in the AD2 treatment (189.20 eggs/female; paired *t*-test, *t* = -0.02, *df* = 4, *P* < 0.05; Table 5).

**Table 4:** Developmental duration of immature F2 *Mallada basalis* fed on two artificial diets.

Diet		Duration of development(d)			
	Egg	1st instar	2nd instar	3rd instar	Pupa
AD1	3.28 ± 0.06b	4.85 ± 0.24a	5.38 ± 0.52b	6.32 ± 0.64a	11.44 ± 0.73a
AD2	4.52 ± 0.11a	5.00 ± 0.50a	9.00 ± 0.90a	8.64 ± 0.78a	12.67 ± 0.56a

Means (± SE) within a column followed by the same letter do not differ significantly (paired *t*-test; *P* > 0.05). Acronyms: AD1, artificial diet 1, and AD2, artificial diet 2

**Table 5:** Reproduction and oviposition parameters of the F2 adult progeny of *Mallada basalis* on two artificial diet treatments.

Parameter	Diet	
	AD1	AD2
Preoviposition period (d)	10.80 ± 1.50a	13.80 ± 4.75a
Oviposition period (d)	31.00 ± 6.15a	20.80 ± 4.60b
Female longevity (d)	40.71 ± 5.10a	36.00 ± 4.13a
Average longevity (d) of females plus males	37.38 ± 4.97a	39.38 ± 6.76a
Female proportion	0.55 ± 0.09a	0.75 ± 0.14b
Daily oviposition (eggs/female/day)	15.56 ± 3.24a	6.80 ± 2.43b
Female oviposition (eggs/female)	456.40 ± 34.11a	189.20 ± 76.76b

Means (± SE) followed by the same letter within a row do not differ significantly (paired *t*-test; *P* >0.05). Acronyms: AD1, artificial diet 1, and AD2, artificial diet 2

The F2 pupation rate in the AD1 treatment (56.00%) was significantly greater than that in the AD2 treatment (24.00%; paired *t*-test, *t* = 2.30, *df* = 4, *P* < 0.05). The F3 egg hatching rate from AD1 (85.00%) was significantly greater than that from AD2 (67.00%; paired *t*-test, *t* = 7.06, *df* = 4, *P* < 0.05). There was no significant difference in the F2 emergence rate between the two diet treatments (paired *t*-test, *t* = 1.73, *df* = 3, *P* = 0.18; S2 Fig).

## Conclusions

This study aimed to improve an artificial diet based on that formulated by Ye et al. [19] for mass rearing the economically important chrysopid predator *M*. *basalis*. When evaluating an artificial diet for the rearing of arthropod natural enemies, it is important to consider development and reproduction of the predator species [25]. In this study, we evaluated the suitability of the artificial diets with respect to development, survival, longevity, and reproductive performance of *M*. *basalis*. Nutritional imbalances within a diet may be expressed only in subsequent generations [26]; therefore, the development and reproductive performance of *M*. *basalis* fed AD1 and AD2 was assessed over two generations, F1 and F2. We found that *M*. *basalis* was able to develop and reproduce when fed artificial diets AD1 and AD2. However, the F1 adult longevity and F2 oviposition period in the AD1 treatment was significantly longer than that in the AD2 treatment. The F1 female proportion, F1 daily oviposition, F1 female oviposition, F2 daily oviposition, and F2 female oviposition from AD1 was significantly larger, while the F1 emergence rate, F2 pupation rate, and F3 egg hatching rate from AD1 was significantly greater compared with those from AD2. These results suggested that AD1 was superior to AD2 in supporting development and reproduction. As for *M*. *basalis* fed AD2, the duration of F2 2nd and 3rd larvae were significantly longer than for F1. The F2 oviposition period was significantly shorter than that of F1, and the F2 female oviposition was significantly less than that of F1. These results indicated that the offspring of *M*. *basalis* fed AD2 were less thrifty. Only diet AD1 contained sucrose and Vitamin C, which suggests that these ingredients may be important nutrient sources leading to the superiority of AD1. The nutrient balance was considered to be more important than the actual amount [22].

The duration of larval developmental of *M*. *basalis* reared on AD1 was longer than that previously found when reared on *C*. *cephalonica* eggs [20], but was similar to the period when reared on other natural prey species such as *Icerya aegyptiaca*, *Ferrisia virgata* and *Planococcus citri* [6, 20]. The pupal duration of *M*. *basalis* reared on AD1 was similar to that of *M*. *basalis* reared on *C*. *cephalonica* eggs, but was longer than of those reared on *I*. *aegyptiaca*, *F*. *virgata* and *P*. *citri*. The adult longevity of *M*. *basalis* reared on AD1 was longer than of that reared on *C*. *cephalonica* eggs, *I*. *aegyptiaca*, *F*. *virgata* or *P*. *citri*. The emergence rate of *M*. *basalis* fed AD1 was greater than that fed on *C*. *cephalonica* eggs, *I*. *aegyptiaca*, *F*. *virgata* or *P*. *citri*. The female proportion of *M*. *basalis* fed AD1 was greater than those recorded on *I*. *aegyptiaca*, *F*. *virgata*, and *P*. *citri*, but was similar to that recorded on *C*. *cephalonica* eggs. *M*. *basalis* fed on AD1 produced more eggs than when fed on the four natural prey species above [6, 20]. However, female *M*. *basalis* had lower fecundity in the present study compared with those of *C*. *septempunctata* fed on an artificial diet containing pork, liver, whole hen’s eggs, brown sugar, vitamins, and preservatives [27]. This may be explained by different levels of fecundity for different species, or perhaps the nutritional quality of the immature and adult diets of *C*. *septempunctata* are superior to our *M*. *basalis* diets. Pork, liver, whole hen’s eggs, brown sugar, and vitamins are important nutrient sources. The increased fecundity could also be attributed to a higher conversion efficiency.

The developmental duration of F1 immature *M*. *basalis* reared on AD1 was longer than that reared on an artificial diet consisting of the same ingredients but with no seawater spirulina [19]. A high content of the dietary alga is suggested to support good larval growth and reduce developmental time [22]. Perhaps the spirulina content in the current study was not high enough. However, the F1 oviposition period and female longevity of *M*. *basalis* in the AD1 treatment was longer in the present study compared with that fed the artificial diet described above [19]. The daily oviposition and female oviposition of F1 from AD1 was larger, while the F2 egg hatching rate from AD1 was greater compared with that from the artificial diet excluding seawater spirulina [19]. The spiral-alga powder was added as a dietary protein source because of its high protein content [22].

*M*. *basalis* was successfully reared on a microcapsulated artificial diet [24]. The fecundity and longevity of *M*. *basalis* in the AD1 treatment was similar to that when reared on an artificial diet consisting of beer yeast, hydrolyzed yeast, casein protein, propionic acid, honey, bee larvae, sucrose, and egg yolk [24]. The hatchability from AD1 was lower than that from the diet of Lee [24], however, the bee larvae in the diet of Lee is not readily available, resulting in poor reliability for the continuous production of the artificial diet.

In summary, the artificial diet AD1 supported development and reproduction of *M*. *basalis* better than the diet formulated by Ye et al. [19] for the mass rearing of this economically important biological control agent. The effect of the quality of AD1 on the long-term rearing of predatory lacewings needs to be further explored as this is a key factor affecting the growth, development, and reproduction of *M*. *basalis*.

## Supporting information

S1 Fig Developmental parameters of immature F1 *Mallada basalis* fed on two artificial diets. (TIF)

S2 Fig Developmental parameters of immature F2 *Mallada basalis* fed on two artificial diets. (TIF)

S1 Table Composition of a previously formulated diet and two modified diets for rearing larvae of the chrysopid *Mallada basalis*. (DOC)

S2 Table Developmental duration of the immature stages of F1 generation *Mallada basalis* fed on two artificial diets. (DOC)

S3 Table Reproduction and oviposition parameters of the F1 adult progeny of *Mallada basalis* fed two artificial diets. (DOC)

S4 Table Developmental duration of the immature stages of F2 *Mallada basalis* fed on two artificial diets. (DOC)

S5 Table Reproduction and oviposition parameters of F2 adult progeny of *Mallada basalis* on two artificial diets. (DOC)
