# Supplementary figures and images for: Rearing of Mallada basalis (Neuroptera: Chrysopidae) on modified artificial diets
Source: PLoS One. 2017 Sep 29;12(9):e0185223. doi: 10.1371/journal.pone.0185223 (PMC5621682; doi:10.1371/journal.pone.0185223)

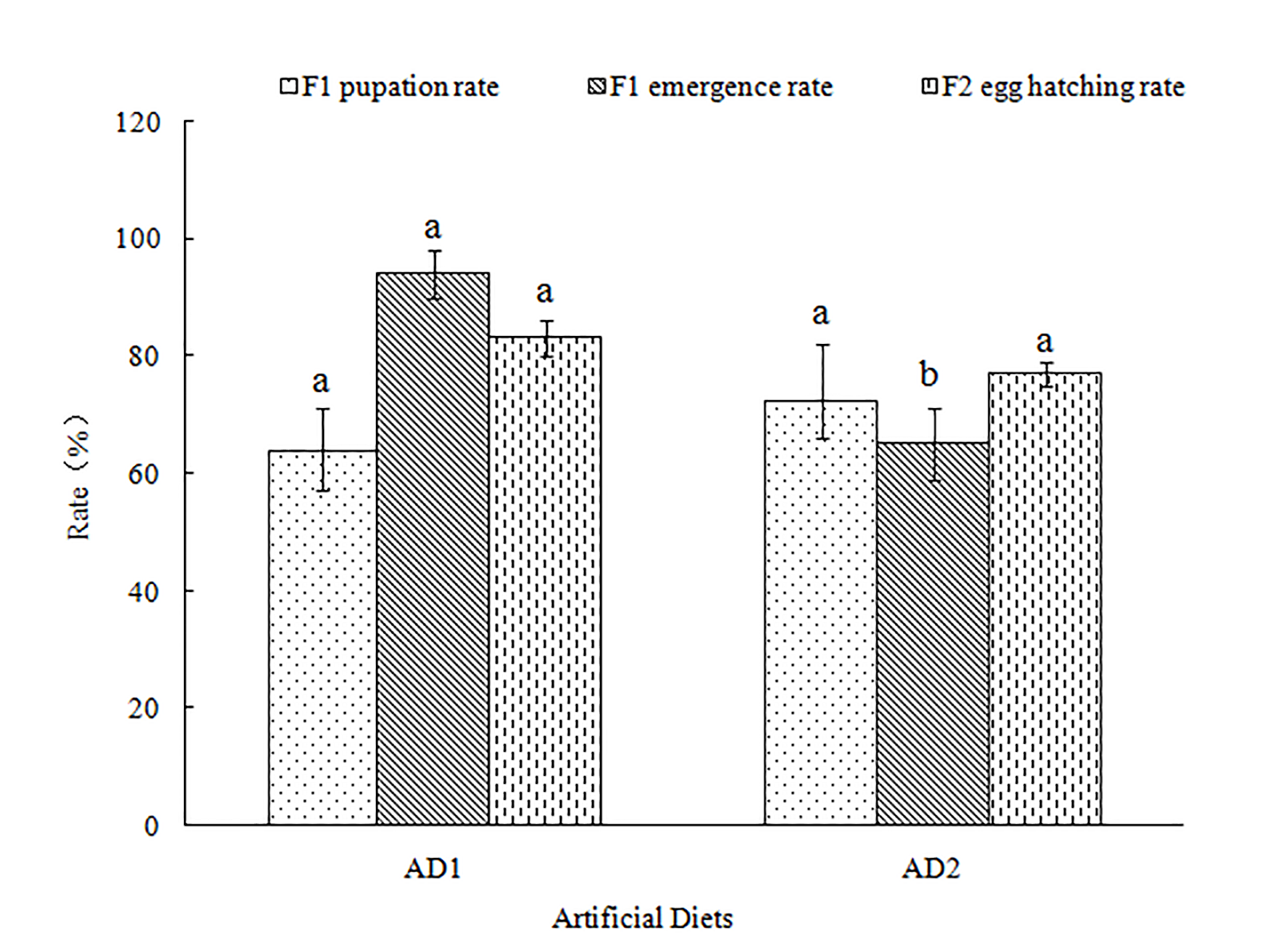

Supplement: S1 Fig — (TIF) [file pone.0185223.s001.tif]

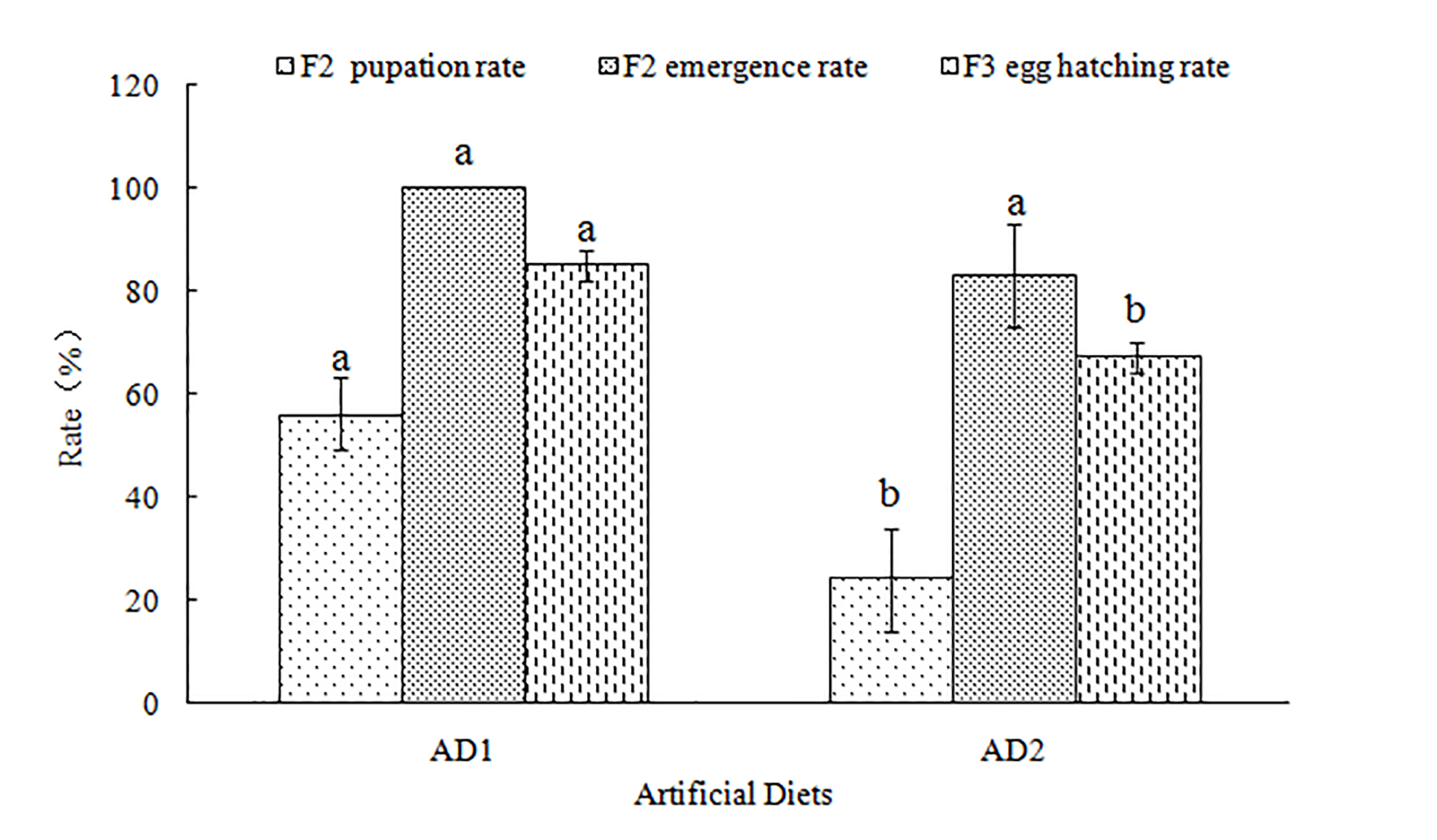

Supplement: S2 Fig — (TIF) [file pone.0185223.s002.tif]
