# Supplementary material for: Rearing of Mallada basalis (Neuroptera: Chrysopidae) on modified artificial diets
Source: PLoS One. 2017 Sep 29;12(9):e0185223. doi: 10.1371/journal.pone.0185223 (PMC5621682; doi:10.1371/journal.pone.0185223)
Supplement: S1 Table — (DOC) [file pone.0185223.s003.doc]

**S1 Table. Composition of three diets for rearing larvae of the chrysopid *Mallada basalis***

| Ingredients | Ingredient amount (g) | | |
| --- | --- | --- | --- |
|  | AD0 | AD1 | AD2 |
| Chicken egg yolk | 40 | 40 | 40 |
| Beer yeast powder | 30 | 30 | 30 |
| Honey | 20 | 20 | 20 |
| Sucrose | 9 | 9 | 0 |
| Trehalose | 1 | 1 | 1 |
| Seawater spirulina | 0 | 1 | 1 |
| Vitamin C | 0.1 | 0.1 | 0 |
| Potassium sorbate | 0.1 | 0.1 | 0.1 |

Acronyms: AD0, previous artificial diet, AD1, artificial diet 1, and AD2, artificial diet 2
