# Supplementary material for: Rearing of Mallada basalis (Neuroptera: Chrysopidae) on modified artificial diets
Source: PLoS One. 2017 Sep 29;12(9):e0185223. doi: 10.1371/journal.pone.0185223 (PMC5621682; doi:10.1371/journal.pone.0185223)
Supplement: S2 Table — (DOC) [file pone.0185223.s004.doc]

**S2 Table. Developmental duration of the immature stages of F1 generation *Mallada basalis*** fed on two artificial diets

| Diet | Duration of development (d) | | | |
| --- | --- | --- | --- | --- |
|  | 1st instar | 2nd instar | 3rd instar | Pupa |
| AD1 | 3.94 ± 0.13b | 4.27 ± 0.16b | 4.62 ± 0.32a | 9.25 ± 0.20b |
| AD2 | 4.54 ± 0.21a | 4.70 ± 0.23a | 4.92 ± 0.29a | 10.46 ± 0.23a |

Means (± SE) within a column followed by the same letter do not differ significantly (paired *t*-test; *P* > 0.05). Acronyms: AD1, artificial diet 1, and AD2, artificial diet 2
