# Supplementary material for: Rearing of Mallada basalis (Neuroptera: Chrysopidae) on modified artificial diets
Source: PLoS One. 2017 Sep 29;12(9):e0185223. doi: 10.1371/journal.pone.0185223 (PMC5621682; doi:10.1371/journal.pone.0185223)
Supplement: S3 Table — (DOC) [file pone.0185223.s005.doc]

**S3 Table. Reproduction and oviposition parameters of the F1 adult progeny of *Mallada basalis*** on the two artificial diet treatments

| Parameter | Diet | |
| --- | --- | --- |
|  | AD1 | AD2 |
| Preoviposition period (d) | 7.57 ± 1.25b | 12.14 ± 1.87a |
| Oviposition period (d) | 32.86 ± 6.07a | 31.57 ± 6.05a |
| Female longevity (d) | 45.40 ± 13.54a | 49.20 ± 6.24a |
| Average longevity of females plus males (d) | 44.57 ± 7.24a | 30.74 ± 5.99b |
| Female proportion | 0.56 ± 0.06a | 0.36 ± 0.05b |
| Daily oviposition (eggs/female/day) | 17.57 ± 3.74a | 8.69 ± 2.14b |
| Female oviposition (eggs/female) | 481.29 ± 54.40a | 307.14 ± 60.77b |

Means (± SE) followed by the same letter within a row do not differ significantly (paired *t*-test; *P* >0.05). Acronyms: AD1, artificial diet 1, and AD2, artificial diet 2
