# Supplementary material for: Rearing of Mallada basalis (Neuroptera: Chrysopidae) on modified artificial diets
Source: PLoS One. 2017 Sep 29;12(9):e0185223. doi: 10.1371/journal.pone.0185223 (PMC5621682; doi:10.1371/journal.pone.0185223)
Supplement: S4 Table — (DOC) [file pone.0185223.s006.doc]

**S4 Table. Developmental duration of immature F2 *Mallada basalis* fed on tw**o artificial diets

| Diet |  | Duration of development（d） | | | |
| --- | --- | --- | --- | --- | --- |
|  | Egg | 1st instar | 2nd instar | 3rd instar | Pupa |
| AD1 | 3.28 ± 0.06b | 4.85 ± 0.24a | 5.38 ± 0.52b | 6.32 ± 0.64a | 11.44 ± 0.73a |
| AD2 | 4.52 ± 0.11a | 5.00 ± 0.50a | 9.00 ± 0.90a | 8.64 ± 0.78a | 12.67 ± 0.56a |

Means (± SE) within a column followed by the same letter do not differ significantly (paired *t*-test; *P* > 0.05). Acronyms: AD1, artificial diet 1, and AD2, artificial diet 2
