# Supplementary material for: Rearing of Mallada basalis (Neuroptera: Chrysopidae) on modified artificial diets
Source: PLoS One. 2017 Sep 29;12(9):e0185223. doi: 10.1371/journal.pone.0185223 (PMC5621682; doi:10.1371/journal.pone.0185223)
Supplement: S5 Table — (DOC) [file pone.0185223.s007.doc]

**S5 Table. Reproduction and oviposition parameters of the F2 adult progeny of *Mallada basalis*** on two artificial diet treatments

| Parameter | Diet | |
| --- | --- | --- |
|  | AD1 | AD2 |
| Preoviposition period (d) | 10.80 ± 1.50a | 13.80 ± 4.75a |
| Oviposition period (d) | 31.00 ± 6.15a | 20.80 ± 4.60b |
| Female longevity (d) | 40.71 ± 5.10a | 36.00 ± 4.13a |
| Average longevity (d) of females plus males | 37.38 ± 4.97a | 39.38 ± 6.76a |
| Female proportion | 0.55 ± 0.09a | 0.75 ± 0.14b |
| Daily oviposition (eggs/female/day) | 15.56 ± 3.24a | 6.80 ± 2.43b |
| Female oviposition (eggs/female) | 456.40 ± 34.11a | 189.20 ± 76.76b |

Means (± SE) followed by the same letter within a row do not differ significantly (paired *t*-test; *P* >0.05). Acronyms: AD1, artificial diet 1, and AD2, artificial diet 2
